# Supplementary figures and images for: Recombulator-X: A fast and user-friendly tool for estimating X chromosome recombination rates in forensic genetics
Source: PLoS Comput Biol. 2023 Sep 18;19(9):e1011474. doi: 10.1371/journal.pcbi.1011474 (PMC10538763; doi:10.1371/journal.pcbi.1011474)

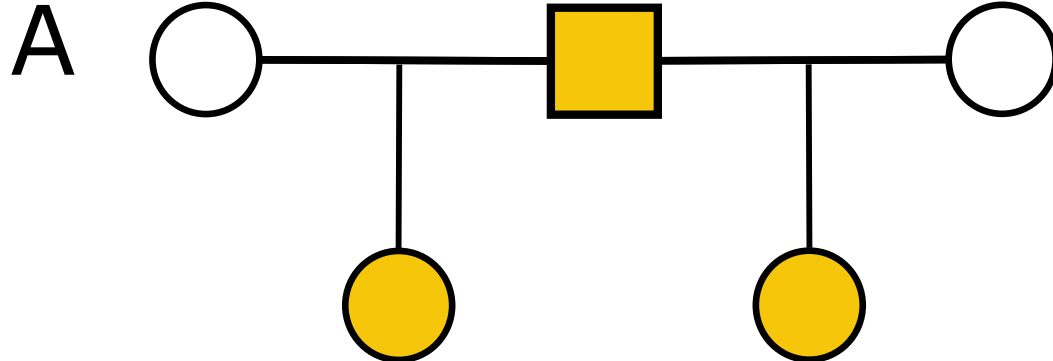

B

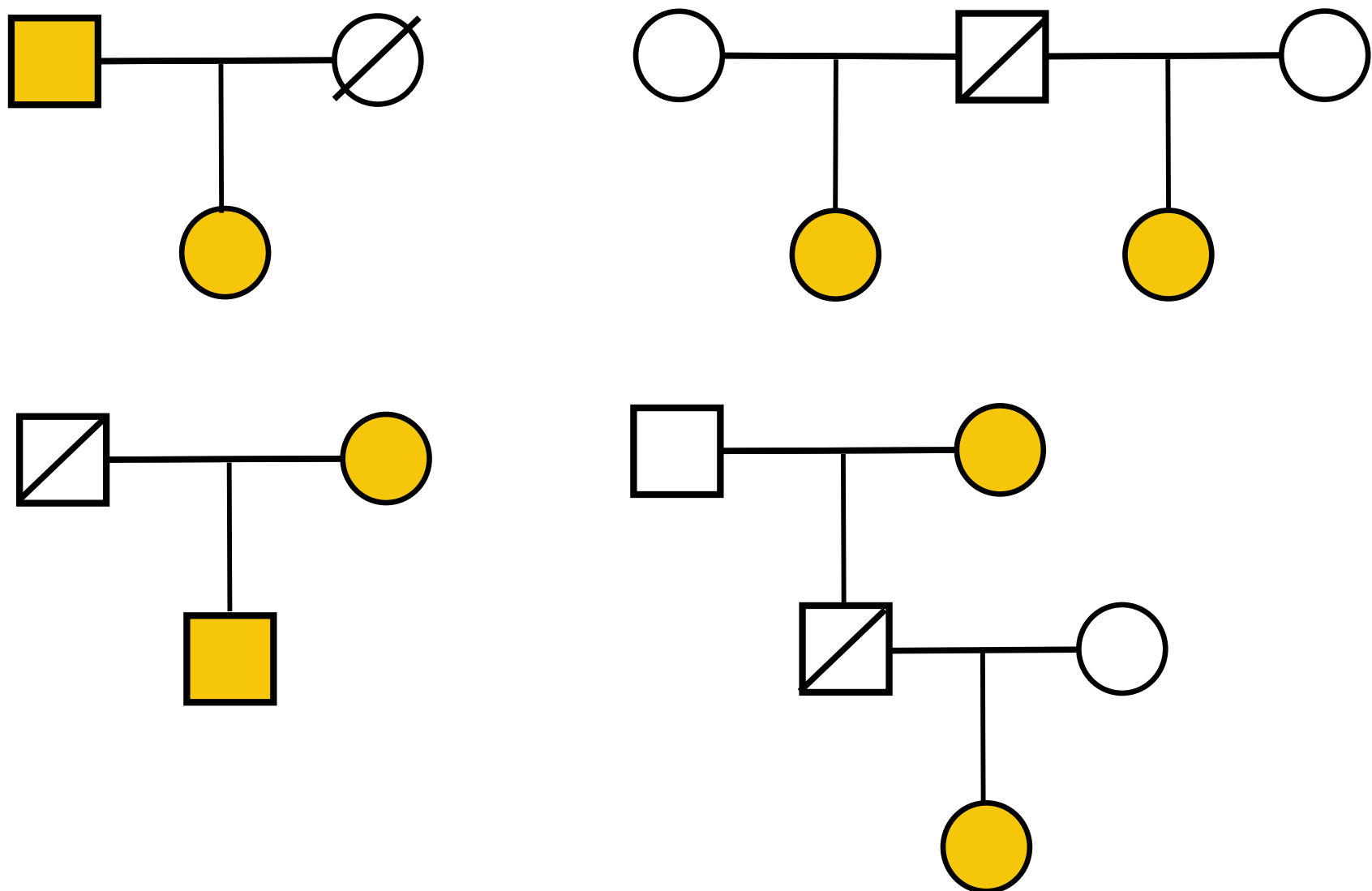

C

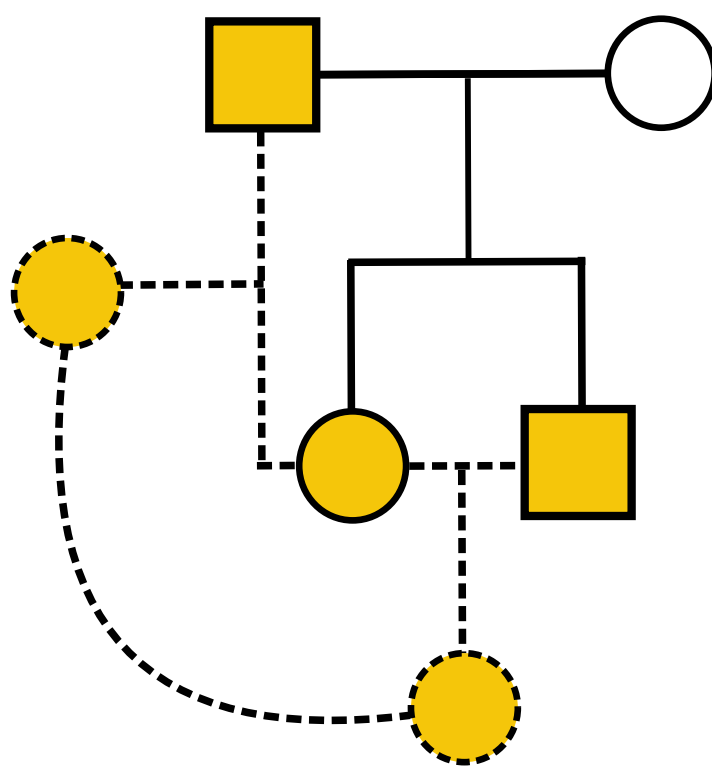

Supplement: S1 Fig — Examples of kinship cases where X chromosomal markers may be informative: half-sisters (A), deficiency paternity test (B) and incest cases (C). The individuals whose genotype needs to be assessed to resolve the kinship case are in yellow. (PDF) [file pcbi.1011474.s002.pdf]

*mother*

STR1: 10

STR2: 5

STR3: 15

STR4: 30

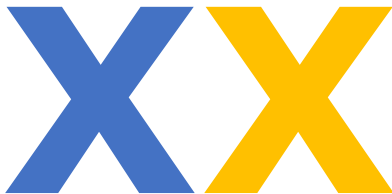

STR1: 9

STR2: 8

STR3: 20

STR4: 32

*son*

STR1: 10

STR2: 8

STR3: 20

STR4: 30

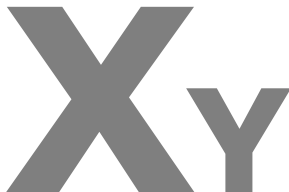

*inheritance vector*

STR1: 10

1

STR2: 8

2

STR3: 20

2

STR4: 30

1

Supplement: S2 Fig — One of the possible inheritance vectors for a given mother-son pair. (PDF) [file pcbi.1011474.s003.pdf]

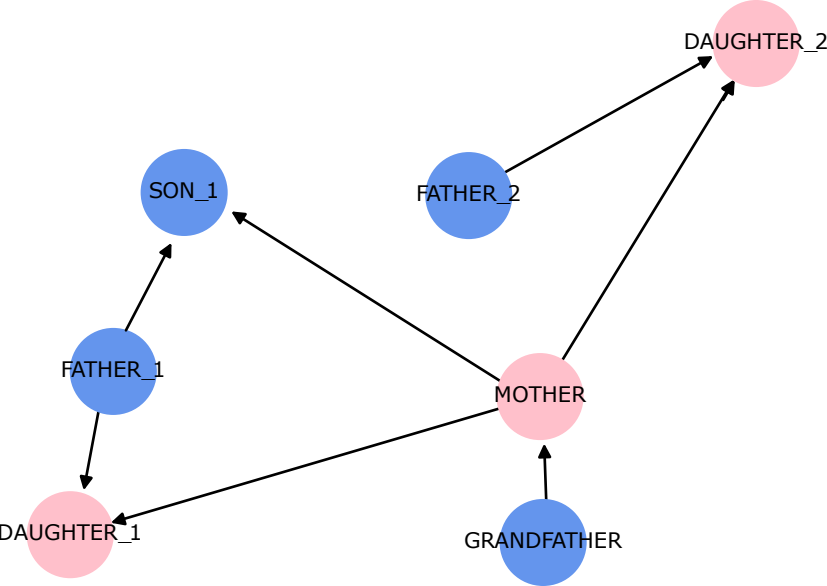

Supplement: S3 Fig — Graph of a simulated family. (PDF) [file pcbi.1011474.s004.pdf]
