# Supplementary material for: Recombulator-X: A fast and user-friendly tool for estimating X chromosome recombination rates in forensic genetics
Source: PLoS Comput Biol. 2023 Sep 18;19(9):e1011474. doi: 10.1371/journal.pcbi.1011474 (PMC10538763; doi:10.1371/journal.pcbi.1011474)
Supplement: S1 Table — Means and standard deviations of the running time needed to compute the likelihood for one family typed over an increasing number of markers. The complete version of this table is reported in S1 File. For each implementation, the number of markers was progressively increased until the computation time went above one second per family. (PDF) [file pcbi.1011474.s005.pdf]

Family Type	type I			
Implementation	direct-loop	direct-numpy	dynamic	dynamic-numba
# of markers				
5	4.18e-04 ± 4.77e-05	1.25e-04 ± 3.15e-05	1.05e-04 ± 1.66e-05	5.21e-06 ± 2.05e-06
10	2.11e-02 ± 3.09e-03	8.27e-04 ± 2.42e-04	1.49e-04 ± 2.50e-05	5.99e-06 ± 2.59e-06
12	9.36e-02 ± 8.81e-03	2.73e-03 ± 9.09e-04	1.75e-04 ± 3.44e-05	5.40e-06 ± 4.04e-06
15	8.86e-01 ± 7.29e-02	2.58e-02 ± 7.90e-03	1.82e-04 ± 1.86e-05	5.06e-06 ± 2.85e-06
20	-	1.54e+00 ± 3.34e-01	2.13e-04 ± 2.48e-05	9.47e-06 ± 1.31e-05
30	-	-	2.92e-04 ± 6.17e-05	5.60e-06 ± 2.40e-06
10000	-	-	6.90e-02 ± 2.57e-03	4.67e-04 ± 5.28e-05

Family Type	type II			
Implementation	direct-loop	direct-numpy	dynamic	dynamic-numba
# of markers				
5	1.41e-02 ± 1.61e-03	3.49e-04 ± 4.84e-05	7.11e-04 ± 1.60e-04	1.46e-04 ± 2.26e-05
10	-	1.77e-01 ± 1.09e-01	1.54e-02 ± 7.11e-03	7.88e-04 ± 3.11e-04
12	-	3.84e+00 ± 2.37e+00	6.02e-02 ± 2.83e-02	2.61e-03 ± 1.11e-03
15	-	-	3.84e-01 ± 2.48e-01	1.58e-02 ± 1.03e-02
20	-	-	-	6.19e-01 ± 6.00e-01
30	-	-	-	-
10000	-	-	-	-
